# Review of the genus *Isodemis* Diakonoff (Lepidoptera, Tortricidae) from China, with description of three new species

**DOI:** 10.3897/zookeys.77.937

**Published:** 2011-01-26

**Authors:** Yinghui Sun, Houhun Li

**Affiliations:** College of Life Sciences, Nankai University, Tianjin 300071, P. R. China

**Keywords:** Lepidoptera, Tortricidae, *Isodemis*, new species, China

## Abstract

The genus Isodemis Diakonoff, 1952 in China is reviewed, with seven species recognized. Three new species are described: Isodemis quadrata **sp. n.**, Isodemis guangxiensis **sp. n.** and Isodemis hainanensis **sp. n.** The female of Isodemis stenotera Diakonoff, 1983 is described for the first time. Variation within Isodemis illiberalis (Meyrick, 1918) and Isodemis stenotera is briefly discussed. Images of the adults and genitalia are provided, along with a key to the described species.

## Introduction

The genus Isodemis was erected by Diakonoff (1952) for the type species Batodes serpentinana Walker, 1863. It belongs to the tribe Archipini in the subfamily Tortricinae. (Diakonoff (1976, 1983) transferred Tortrix illiberalis Meyrick, 1918 to Isodemis and described Isodemis stenotera from Sumatra. (Razowski (2000, 2009a, 2009b) described Isodemis proxima Razowski, 2000 from Chinese Taiwan, and Isodemis brevicera Razowski, 2009, Isodemis longicera Razowski, 2009 and Isodemis ngoclinha Razowski, 2009 from Vietnam. Currently, Isodemis consists of seven species, mainly distributed in Southeast Asia.

**Map 1.**
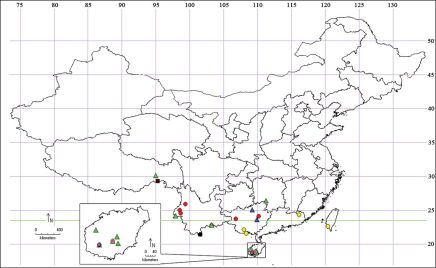
The distribution of Isodemis Diakonoff in China. ● (red) Isodemis illiberalis (Meyrick) ▲ (green) Isodemis stenotera Diakonoff ● (yellow) Isodemis proxima Razowski ● (black) Isodemis serpentinana (Walker) ■ (red) Isodemis quadrata sp. n. ▲ (blue) Isodemis guangxiensis sp. n. ■ (magenta) Isodemis hainanensis sp. n.

Prior to the present study, four species were recorded in China: Isodemis serpentinana (Walker, 1863), Isodemis illiberalis (Meyrick, 1918), Isodemis stenoteraDiakonoff, 1983 and Isodemis proxima Razowski, 2000. The aim of the present paper is to review the genus Isodemis in China and to describe three new species. A key is provided on a worldwide basis based on the forewing patterns and the male genitalia except Isodemis ngoclinha Razowski, 2009 whose male remains unknown. A map is provided to show the distribution of Isodemis species in China (Map 1).

## Material and methods

Examined specimens were collected by light traps. Terminology follows Diakonoff (1948) and Razowski (2009a, b) in descriptions of forewing pattern and genitalia. Genitalia dissection and slide mounting methods follow Li (2002). The examined specimens, including the types of the new species, are deposited in the Insect Collection, College of Life Sciences, Nankai University, Tianjin, China.

## Taxonomic accounts

### Isodemis

Diakonoff, 1952

Isodemis Diakonoff 1952: 147. Type species: Batodes serpentinana Walker, 1863 (original designation).

#### Diagnosis.

Isodemis is characterized by the labial palpus obliquely uprising almost as high as upper edge of eye; the forewing dominantly yellowish brown or ochreous brown; the median fascia interrupted or indistinct near costal margin; male genitalia with gnathos hooked, valva with a C-shaped plica, with numerous fine wrinkles between plica and costa, and the sacculus with terminal process; female genitalia with the ductus bursae usually with cestum, and the single dentate signum with a conspicuous globular process placed posteriorly in the corpus bursae.

#### Distribution.

China, Vietnam, Thailand, Indonesia, Nepal, India and Sri Lanka.

#### Discussion.

Most species of this genus show a stong sexual dimorphism, which makes species identification difficult.Of the seven previously described species, Isodemis longicera Razowski, 2009 and Isodemis brevicera Razowski, 2009 were described from the males, while Isodemis ngoclinha Razowski, 2009 was described from females. Currently, no additional knowledge has been added to these three species. Therefore, we have excluded Isodemis ngoclinha from the key based on forewing patterns and male genitalia.

#### Key to the known species of Isodemis.

**Table:** 

1	Forewing with a semicircular pattern above dorsum	2
–	Forewing without such a pattern above dorsum	4
2	Subapical blotch extending from costal margin to tornus	Isodemis illiberalis (Meyrick, 1918)
–	Subapical blotch not reaching tornus	3
3	Uncus broad rectangular, phallus with one cornutus in male genitalia	Isodemis quadrata sp. n.
–	Uncus broadened slightly from basal 1/4 to apex, phallus with two cornuti in male genitalia	Isodemis guangxiensis sp. n.
4	Forewing with an annular pattern at middle	5
–	Forewing without such a pattern at middle	7
5	Terminal process of sacculus long, reaching plica in male genitalia	Isodemis longicera Razowski, 2009
–	Terminal process of sacculus short, not reaching plica in male genitalia	6
6	Uncus with basal portion narrower than distal portion	Isodemis brevicera Razowski, 2009
–	Uncus nearly rectangular, slightly narrowed in distal 2/5	Isodemis hainaniensis sp. n.
7	Phallus with one cornutusin male genitalia	Isodemis proxima Razowski, 2000
–	Phallus with two cornutiin male genitalia	8
8	Cornuti equal in length, not undulate	Isodemis stenotera Diakonoff, 1983
–	Cornuti unequal in length, longer one undulate	Isodemis serpentinana (Walker, 1863)

### Isodemis illiberalis

(Meyrick, 1918)

Figs 1 11–12

Tortrix illiberalis Meyrick 1918: 168. Type locality: India.
Cacoecia interjecta Meyrick 1922: 496. Type locality: India.
Syndemis montivola Diakonoff 1941: 40. Type locality: India.
Isodemis illiberalis (Meyrick 1918): Diakonoff 1976: 113.

#### Material examined.

1 ♂, **China, Guangxi Zhuang Autonomous Region:** Milv Village, Nanping Town, Shangsi County (22°09'N, 107°58'E), 770 m, 3.IV.2002, coll. Shulian Hao and Huaijun Xue; 1 ♂, Mt. Pinglong, Shangsi County (22°09'N, 107°58'E), 510 m, 6.IV.2002, coll. Shulian Hao and Huaijun Xue; 1 ♂, Mt. Villa Huawang, Jinxiu Yao Autonomous County (24°08'N, 110°11'E), 550 m, 14.IV.2002, coll. Shulian Hao and Huaijun Xue; 8 ♂♂, Grand Canyon Laohutiao, Napo County (23°44'N, 106°48'E), 30.VII.2008, coll. Liusheng Chen and Guoyi Wu; 1 ♂, **China, Yunnan Province:** Tengchong County (25°01'N, 98°30'E), 1950 m, 28.IX.2002, coll. Huaijun Xue; 2 ♂♂, Xiaoheishan Nature Reserves (24°35'N, 98°41'E), 2300 m, 10.VIII.2005, coll. Yingdang Ren.

#### Diagnosis.

**Figures 1–10.**
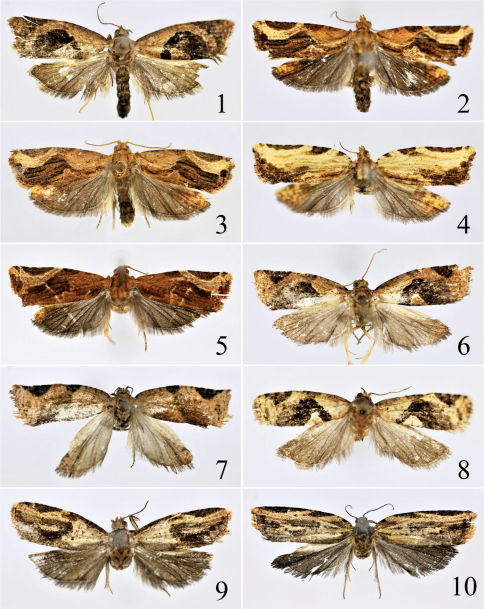
Adults of Isodemis spp. **1** Isodemis illiberalis (Meyrick), ♂ **2–3** Isodemis stenotera Diakonoff, ♂ (showing variation of markings) **4** Isodemis stenotera Diakonoff, ♀ **5** Isodemis proxima Razowski,♂ **6** Isodemis quadrata sp. n., holotype, ♂ **7** Isodemis quadrata sp. n., paratype, ♀ **8** Isodemis guangxiensis sp. n. (male) **9** Isodemis hainanensis sp. n., holotype, ♂ **10** Isodemis hainanensis sp. n., paratype, ♀.

**Figures 11–15.**
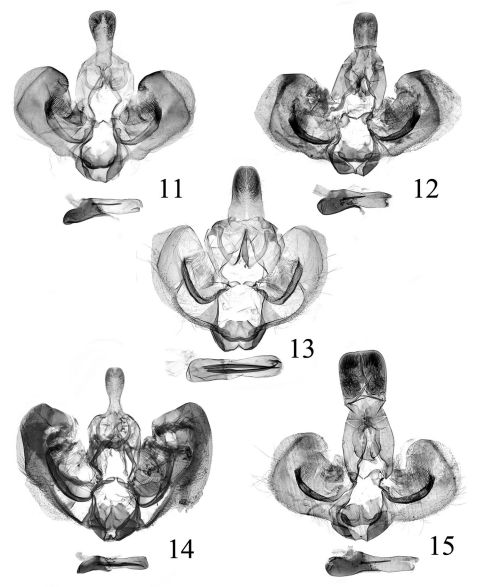
Male genitalia of Isodemis spp. **11–12** Isodemis illiberalis (Meyrick): **11** slide no. SYH10014 **12** slide no. SYH10002 (showing variation of genitalia) **13** Isodemis stenotera Diakonoff, slide no. SYH09017 **14** Isodemis proxima Razowski,slide no. SYH10015 **15** Isodemis quadrata sp. n., holotype, slide no. WXP03332.

Adult (Fig. 1) with wingspan 16.0–19.5 mm. This species is characterized by the male genitalia with the uncus broadening from basal 1/3 to blunt apex, and the phallus having eight to twenty-three deciduous cornuti and a single non-deciduous cornutus (Figs 11–12); in the female genitalia by the sterigma deeply V-shaped, the ductus bursae about 1.5 times the corpus bursae, and the globular process almost 1/2 length of the signum (Diakonoff 1941: 40, fig. 5). It can be easily distinguished from its congeners by the median fascia extending from below distal half of the costal fold to the dorsum and the subapical blotch reaching the tornus.

#### Distribution.

China (Guangdong, Guangxi, Yunnan), Vietnam, Thailand, India, Nepal.

#### Variation.

The uncus broadens from basal 1/3 to apex and the phallus has sixteen to twenty-three deciduous cornuti and a single non-deciduous cornutus in the specimens collected in Guangxi (Fig. 11); while the uncus is parallel sided and the phallus bears eight deciduous cornuti and a single non-deciduous cornutus in the specimens collected in Yunnan (Fig. 12).

### Isodemis stenotera

Diakonoff, 1983

Figs 2–4 13 18

Isodemis stenotera Diakonoff 1983: 92. Type locality: Indonesia.

#### Material examined.

1 ♂, 1 ♀, **China, Hainan Province:** 12–15.V.2005, coll. Min Wang and Liusheng Chen; 6 ♂♂, 2 ♀♀, Jianfengling (18°44'N, 109°10'E), V.2004, coll. Min Wang *et al.*; 1 ♂, 1 ♀, (21.V.2004) and 6 ♂♂, Jianfengling (18°44'N, 109°10'E), 940 m, 4–7.VI.2007, coll. Zhiwei Zhang and Weichun Li; 4 ♂♂, 1 ♀, Tianchi, Jianfengling, 790–810 m, 30.III-1.IV.2008, coll. Bingbing Hu and Haiyan Bai; 3 ♂♂, 20.IV.-12.VI.2010, coll. Bingbing Hu and Jing Zhang; 2 ♂♂, Yinggeling (19°02'N, 109°50'E), 28.VIII-3.IX.2005, coll. Min Wang and Liusheng Chen; 1 ♂, Yinggeling, 620 m, 28.IV.2010, coll. Bingbing Hu and Jing Zhang; 3 ♂♂, 1 ♀, Mt. Wuzhi (18°52'N, 109°40'E), 700 m, 18–19.V.2007, coll. Zhiwei Zhang and Weichun Li; 7 ♂♂, 740 m, 14–15.IV.2009, coll. Qing Jin and Bingbing Hu; 2 ♂♂, Shuiman Town, Mt. Wuzhi (18°52'N, 109°40'E), 630 m, 16.IV.2009, coll. Qing Jin and Bingbing Hu; 1 ♀, Mt. Diaoluo (18°47'N, 109°52'E), 940 m, 2.VI.2007, coll. Zhiwei Zhang and Weichun Li; 1 ♂, 1 ♀, River Nancha, Bawangling (19°16'N, 109°03'E), 600 m, 9.VI.2007, coll. Zhiwei Zhang and Weichun Li; 9 ♂♂, 4 ♀♀ (4–9.IV.2008, coll. Bingbing Hu and Haiyan Bai); 3 ♂♂, 2 ♀♀ (23.IV.2009, coll. Bingbing Hu and Qing Jin), Dong’er Workstation, Bawangling (19°16'N, 109°03'E), 1000 m; 3 ♂♂, Dongyi Protection Station, Bawangling (19°16'N, 109°03'E), 650 m, 7.IV.2008, coll. Bingbing Hu and Haiyan Bai; **China, Hunan Province:** 3 ♂♂, Mt. Shunhuang, Dong’an County (26°24'N, 111°17'E), 20–22.V.2007, coll. Min Wang and Liusheng Chen; **China, Yunnan Province:** 1 ♂, Rare Botanic Garden, Ruili City (24°00'N, 97°50'E), 1000 m, 6.VIII.2005, coll. Yingdang Ren.

#### Diagnosis.

This species is very similar to the type species Isodemis serpentinana both in appearance and in the genitalia,but can be distinguished by the male genitalia having two nearly straight cornuti that are equal in length, and the female genitalia having the ductus bursae about the same length as the corpus bursae and broadening slightly from the inception of the ductus seminalis to the corpus bursae. In Isodemis serpentinana, the male genitalia have a phallus bearing two cornuti that are unequal in length with the longer one undulate,and the female genitalia have a ductus bursae about 1.5 times the length of the corpus bursae and slightly broader from middle of the ductus seminalis to corpus bursae (Diakonoff 1948: 511, fig. 37).

#### Description.

##### Adult: Male

(Figs 2–3) wingspan17.5–22.0 mm. Female (Fig. 4) wingspan 19.0–27.0 mm. Head, antenna and labial palpus yellow, scattered with ochreous. Thorax and tegula ochreous brown tinged with yellow. Forewing broad, nearly rectangular, apex slightly protruding anteriorly; ground color yellow, with scattered pale ochreous scales medially, densely covered with ochreous brown scales along dorsal area; markings ochreous brown with sparse brownish black scales: costal margin with two dots near base, with a triangular spot at basal 1/5; median fascia interrupted or indistinct medially, extending to distal 1/3 of dorsum, then along tornus obliquely reaching anteriorly to middle of termen; faint pale ochreous yellow stripe from below costal portion of median fascia to termen below apex, gradually narrowing; subapical blotch from middle of costal margin to before apex, narrowly stripe-shaped, with brownish black and yellow dots along costal margin; cilia ochreous mixed with brownish black, yellow at apex. Hindwing pale grayish brown, distally with a large yellow patch tinged with sparse pale ochreous brown scales; cilia pale grayish brown. Legs pale yellow, mixed with brownish black on ventral side of foreleg; outer side of mid- and hindlegs yellow, tinged with brownish black. Abdomen grayish brown.

**Figures 16–20.**
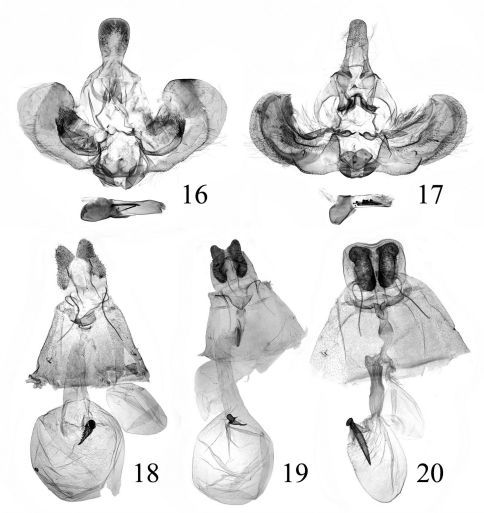
Genitalia of Isodemis spp. 16–17. ♂: **16** Isodemis guangxiensis sp. n., paratype, slide no. SYH10010 **17** Isodemis hainanensis sp. n., holotype, slide no. SYH09042 18–20. ♀: **18** Isodemis stenotera Diakonoff, slide no. SYH10005 **19** Isodemis quadrata sp. n., paratype, slide no. SYH10003 **20** Isodemis hainanensis sp. n. paratype, slide no. SYH09043.

##### Female genitalia

(Fig. 18). Papilla analis long and narrow, distal 2/5 slightly expanded. Apophysis anterioris about 1.3 times length of apophysis posterioris. Sterigma nearly band-shaped, protrudent backward posterolaterally. Antrum short, with inner sclerite anteriorly; ductus seminalis from posterior 1/5 of ductus bursae; ductus bursae about same length as corpus bursae, broadened slightly from inception of ductus seminalis to corpus bursae; cestum absent. Corpus bursae rounded; signum horn-shaped, with tiny spines on ventral surface, dentate marginally, its globular process about 1/4 length of signum.

#### Distribution.

China (Hunan, Guangxi, Hainan, Tibet, Yunnan), Indonesia (Sumatra).

#### Variation.

In some male individuals, the median fascia has a small brownish yellow spot placed near the posterior 1/4 of termen, connected with the yellow stripe above it (Fig. 3); the vinculum has a tiny spine at middle on the anterior margin in the male genitalia.

#### Notes.

The female of this species is described for the first time.

### Isodemis proxima

Razowski, 2000

Figs 5 14

Isodemis proxima Razowski 2000: 325. Type locality: Taiwan.

#### Material examined.

1 ♂, **China, Guangxi Province:** Mt. Pinglong, Shangsi County (22°09'N, 107°58'E), 250 m, 7.IV.2002, coll. Shulian Hao and Huaijun Xue; 1 ♂, Dongzhong Woodfarm, Fangchenggang City (21°37'N, 108°20'E), 370 m, 9.IV.2002, coll. Shulian Hao and Huaijun Xue; 3 ♂♂, **China, Hainan Province:** Shuiman Town, Mt. Wuzhi (18°52'N, 109°40'E), 650 m, 15–17.V.2007, coll. Zhiwei Zhang and Weichun Li; 4 ♂♂, Shuiman Town, Mt. Wuzhi (18°52'N, 109°40'E), 630–740 m, 13–17.IV.2009, coll. Qing Jin and Bingbing Hu.

#### Diagnosis.

Adult (Fig. 5) with wingspan 16.0–21.0 mm. This species is very similar to Isodemis stenotera bothin appearance and in the genitalia, but can be distinguished by the forewing dominantly dark ochreous brown; the uncus narrowed basally and the phallus with single cornutus in male genitalia (Fig. 14); and the ductus bursae about 1.5 times length of the corpus bursae in female genitalia (Razowski 2000: 325, fig. 14). In Isodemis stenotera,the forewing is mainly ochreous brown; the uncus is broadenedbasally and the phallus bears two cornuti; and the ductus bursae ia about the same length as the corpus bursae.

#### Distribution.

China (Guangdong, Guangxi, Hainan, Taiwan).

### Isodemis serpentinana

(Walker, 1863)

Batodes serpentinana Walker 1863: 317. Type locality: Indonesia.
(Tortrix?) sulana Walker 1866: 1784. Type locality: New Guinea.
Cacoecia serpentinana  (Walker, 1863): Meyrick 1912: 18.
Tortrix serpentinana  (Walker, 1863): Meyrick 1921: 149.
Syndemis serpentinana  (Walker, 1863): Diakonoff 1941: 41.
Isodemis serpentinana  (Walker, 1863): Diakonoff 1952: 147.

#### Distribution.

China (Hainan, Yunnan, Taiwan); India, Indonesia (Borneo, Java, Sumatra), New Guinea, Philippine, Sri Lanka, Tailand.

#### Notes.

Based on the description of Isodemis serpentinana and the illustration of its male genitalia provided by Diakonoff (1941), this species is distinguished by the phallus bearing two unequal cornuti with the longer one undulate in the male genitalia. When Diakonoff (1983) described Isodemis stenotera, hepointed out that Isodemis stenotera was very similar to Isodemis serpentinana superficially, but could be separated by the male genitalia having two cornuti equal in length. By checking the holotype deposited in the Natural History Museum, London, we also found that the subapical blotch in Isodemis serpentinana is subtriangular, while it is narrowly semioval in Isodemis stenotera. More differences of the two species are stated under Isodemis stenotera.

### Isodemis quadrata sp. n.

urn:lsid:zoobank.org:act:8BBC12C8-21E6-4349-9EA1-D0254A20E481

Figs 6–7 15 19 

#### Type material.

Holotype ♂ – **China**, **Xizang (Tibet) Autonomous Region:** Hanmi, Medog County (29°13'N, 95°18'E), 2380 m, 9.VIII.2003, coll. Xinpu Wang and Huaijun Xue, genitalia slide no. WXP03332. Paratypes: 1 ♂, 2 ♀♀, same data as for holotype.

#### Diagnosis.

The male is similar to Isodemis illiberalis superficially, but can be separated by the median fascia extending from the costal margin to the dorsum, the subapical blotch reaching across 1/3 width of wing and the quadrate uncus. In Isodemis illiberalis, the median fascia extends from the lower edge of costal fold to the dorsum, the subapical blotch reaches the tornus, and the uncus broadens from the basal 1/3 to the apex. The female genitalia are close to those of Isodemis stenotera, but can be distinguished by the ductus bursae being longer than the corpus bursae and the signum without tiny spines on its ventral surface; in Isodemis stenotera, the ductus bursae is about the same length as the corpus bursae and the signum consists of tiny spines on its ventral surface. In addition, the female can be easily separated from Isodemis stenotera by the ground color being yellowish brown mixed with ochreous, the median fascia extending from the costal margin to distal 1/3 of the dorsum, and the subapical blotch nearly inverted triangular; in Isodemis stenotera, the ground color is yellow with scattered pale ochreous scales, the median fascia reaches the middle of termen and is interrupted or indistinct medially, and the subapical blotch is narrowly stripe-shaped.

#### Description.

##### Adult: Male

(Fig. 6) wingspan 19.5–20.0 mm. Head, antenna and labial palpus yellowish brown, with scattered ochreous brown. Thorax and tegula ochreous brown mixed with yellowish brown. Forewing yellowish brown with sparse ochreous scales; costal fold tinged with brownish black, about 3/5 length of costal margin; markings brownish black mottled with ochreous: median fascia extending from costal margin to distal 1/3 of dorsum, slightly interrupted near costal fold, with a large rounded gray patch present on its outer side posteriorly; short stripe from basal 1/3 of dorsum extending anteriorly and joining inner side of median fascia at anterior 1/3, forming an irregular semicircular pattern above dorsum, leaving a rounded subtriangular patch of groundcolour at middle of dorsum; subapical blotch inverted triangular, extending from distal 1/3 of costal margin to before apex, reaching across 1/3 width of wing; cilia yellowish brown. Hindwing dark gray, distally with a pale grayish brown patch tinged with yellowish brown; cilia dark gray. Legs dark yellow, tinged with brownish black on ventral side of foreleg and on outer side of mid- and hindlegs. Abdomen grayish brown.

##### Female

(Fig. 7) wingspan 21.0–22.5 mm. Head and labial palpus dark grayish brown. Antenna brownish black tinged with yellowish brown. Forewing broader than in male, nearly rectangular, apex slightly protruding anteriorly; costal margin tinged with brownish black; posterior 4/5 of median fascia ochreous brown with sparse brownish black scales; lower edge of subapical blotch slightly rounded; with small diffused patch placed near middle of termen. Hindwing gray, anterodistally with a yellowish brown patch mottled with brownish black.

##### Male genitalia

(Fig. 15). Tegumen developed. Uncus nearly quadrate, straight on posterior margin. Gnathos arm slender and long; terminal plate nearly triangular, about 1/3 length of arm. Valva with length about 1.5 times width, rounded terminally; transtilla spine-shaped, disconnected medially. Sacculus weakly sclerotized; terminal process nearly thumblike, reaching plica. Vinculum somewhat concave at middle on anterior margin, with two small spines near middle of anterior margin. Juxta approximately oval, slightly concave at middle anteriorly. Phallus slightly longer than length of valva, straight, dilated basally, with ten deciduous cornuti and a single non-deciduous cornutus that is about 1/3 length of phallus.

##### Female genitalia

(Fig. 19). Papilla analis narrow and long. Apophysis anterioris slightly longer than apophysis posterioris. Sterigma inverted subtriangular. Antrum short, with inner sclerite anteriorly; ductus seminalis coming from anterior margin of antrum; ductus bursae longer than corpus bursae, curved perpendicularly at posterior 1/3; cestum placed between posterior 1/3 of ductus bursae and anterior margin of antrum. Corpus bursae rounded; signum horn-shaped, dentate marginally, its globular process about 1/3 length of signum.

#### Distribution.

China (Tibet).

#### Etymology.

The specific name is from the Latin *quadratus* (= square), referring to the rectangular uncus in the male genitalia.

### Isodemis guangxiensis sp. n.

urn:lsid:zoobank.org:act:F2E5B790-7F5B-46F6-813D-F1CC182AD4E6

Figs 8 16 

#### Type material.

Holotype ♂ – **China, Guangxi Zhuang Autonomous Reging:** Rongshui Miao Autonomous County (25°04'N, 109°13'E), 31.VII.2003, genitalia slide no. SYH09041. Paratype: 1 ♂, Huaping Nature Reserves (23°39'N, 109°55'E), 1300 m, 1.VIII.2006, coll. Weichun Li.

#### Diagnosis.

This species is similar to Isodemis illiberalis both in appearance and in male genitalia, but can be separated by the median fascia extending from the costal margin to the dorsum, the subapical blotch reaching across 1/3 width of wing and the phallus having eight deciduous cornuti and two non-deciduous cornuti. In Isodemis illiberalis, the median fascia extends from below the costal fold to the dorsum, the subapical blotch reaches the tornus and the phallus has eight to twenty-three deciduous cornuti and a single non-deciduous cornutus. Isodemis guangxiensis is superficially also similar to Isodemis quadrata, the differences between them are as follows: in Isodemis guangxiensis, the uncus broadens from the basal 1/4 to the apex and the phallus bears eight deciduous cornuti and two non-deciduous cornuti, whereas in Isodemis quadrata, the uncus is quadrate and the phallus has ten deciduous cornuti and a single non-deciduous cornutus.

#### Description.

##### Adult: Male

(Fig. 8) wingspan 18.0–18.5 mm. Head yellowish brown. Antenna and labial palpus ochreous brown, with scattered yellowish brown scales. Thorax and tegula dark grayish brown, sparsely mixed with yellowish brown. Forewing yellowish brown tinged with ochreous scales; costal fold densely suffused with brownish black scales, about 3/5 length of costal margin; markings brownish black mottled with ochreous: median fascia extending from costal margin to distal 2/5 of dorsum, interrupted near costal margin, with nearly oval gray patch present on its outer side posteriorly; short stripe from basal 1/4 of dorsum extending anteriorly and touching inner side of median fascia at anterior 1/3, forming an irregularly semicircular pattern above dorsum, leaving a rounded subtriangular patch of groundcolour near middle of dorsum; subapical blotch inverted triangular, from distal 1/3 of costal margin to before apex, reaching across 1/3 width of wing; with small diffusion near middle of termen; cilia yellowish brown mixed with brownish black. Hindwing and cilia grayish brown. Legs dark yellow, mottled brownish black on ventral side of foreleg and on outer side of mid- and hindlegs. Abdomen grayish brown.

##### Male genitalia

(Fig. 16). Uncus nearly rectangular in basal 1/4, then broadened slightly to rounded apex, densely setose in distal half. Gnathos arm slender and long; terminal plate triangular, about 2/3 length of arm. Valva slightly widened distally, length about 2 times of width, rounded terminally; transtilla irregularly round, with pointed apical process. Sacculus weakly sclerotized, protruding ventrally at middle; terminal process nearly triangular, rounded at apex, reaching plica. Vinculum somewhat concave at middle on anterior margin. Juxta large and broad, straight on anterior margin; posterior margin concave and arched, protruding posterolaterally. Phallus slightly shorter than length of valva, slightly curved and dilated in basal 2/5, with eight deciduous cornuti and two non-deciduous unequal cornuti that are about 1/3 length of phallus.

##### Female.

Unknown.

#### Distribution.

China (Guangxi).

#### Etymology.

The name is derived from the type locality.

### Isodemis hainanensis sp. n.

urn:lsid:zoobank.org:act:0BDC5A29-95B3-43AC-A8B3-77C9BDC4DA8F

Figs 9–10 17 20 

#### Type material.

Holotype ♂ − **China, Hainan Province:** Mt. Wuzhi Nature Reserves (18°52'N, 109°40'E), 740 m, 15.IV.2009, coll. Qing Jin and Bingbing Hu, genitalia slide no. SYH09042. Paratype: 1 ♀, Jianfengling (18°44'N, 109°10'E), 800–900 m, 6.XII.2009, coll. Zhaohui Du.

#### Diagnosis.

This species is superficially very similar to Isodemis longicera and Isodemis brevicera, but it can be separated by the uncus being slightly narrowed in the distal 2/5 and rounded apically, the spine-shaped terminal process of the sacculus not reaching plica and the phallus bearing eight cornuti. In Isodemis longicera, the uncus is slightly concave at middle on posterior margin, the terminal process of the sacculus reaches the plica and the phallus bears twelve cornuti; in Isodemis brevicera, the uncus broadens from base to apex, the terminal process of the sacculus is nearly triangular, and the phallus has ten cornuti. Female genitalia resemble those of Isodemis ngoclinha, but can be distinguished by the anterior portion of the papilla analis not being inflated and the sterigma extending posteriorly uniformly in width to both sides; whereas in Isodemis ngoclinha, the basal 2/3 of papilla analis is inflated, and the sterigma widens from the inception of ductus bursae to the lateral side.

#### Description.

##### Adult: Male

(Fig. 9) wingspan 17.5 mm. Head, antenna and labial palpus grayish brown, mixed with brownish black scales. Thorax and tegula brownish black, with sparse grayish brown scales. Forewing dark yellowish brown, tinged with ochreous scales in distal half; costal fold narrow, about 1/2 length of costal margin, brownish black, with short black stripes along costal margin; markings brownish black mottled ochreous: median fascia interrupted near costal margin, reaching above tornus, then extending obliquely anteriorly to lower corner of cell; longitudinal broad median stripe from apical margin of median fascia to end of cell, forming an annular pattern with indentation; subapical blotch from distal 2/5 of costal margin to apex, reaching across 1/4 width of wing; pale brownish black terminal lines between veins; dorsal area densely suffused with grayish brown scales, forming a broad band along dorsum; cilia grayish brown mixed with brownish black. Hindwing and cilia grayish brown. Legs dark yellowish brown, mottled brownish black on ventral side of foreleg and on outer side of mid- and hindlegs. Abdomen dark grayish brown.

##### Female

(Fig. 10) wingspan 22.0 mm. As in male except with small, brownish black basal fascia near base; costal margin more arched basallythan in male; subapical blotch from middle of costal margin to apex, narrow, reaching across 1/5 width of wing; stripe along anal vein to beyond mid-length of wing.

##### Male genitalia

(Fig. 17). Tegumen with a small triangular process at posterior 1/4 medially. Uncus nearly rectangular, slightly narrowed in distal 2/5, sparsely setose in distal half, rounded at apex. Gnathos arm tapering to distal end, with distinct lateral prominence that bears many tiny spines; terminal plate short, about 1/2 length of arm. Valva length about two times width, rounded terminally; plica extremely thin, straight; small sclerotized projection at basal 1/3 between plica and ventral margin. Transtilla irregularly oval, with pointed and curved apical process. Sacculus sclerotized; terminal process a long spine, not reaching plica. Vinculum sclerotized anteriorly. Juxta approximately semioval, slightly concave at middle on posterior margin. Phallus about 2/3 length of valva, pistol-shaped, basal 1/3 dilated; cornuti composed of one deciduous cornutus and seven non-deciduous cornuti, each about 1/4 length of phallus.

##### Female genitalia

(Fig. 20). Papilla analis broad. Apophysis anterioris about 1.3 times length of apophysis posterioris. Sterigma narrow and long transversely, weakly notched at middle posteriorly. Antrum long, about 1/4 length of ductus bursae, anterior 1/3 sclerotized; ductus bursae slightly longer than corpus bursae, wrinkled; ductus seminalis from middle of ductus bursae; cestum absent. Corpus bursae oval, posterior half wrinkled; signum large spine-shaped, dentate marginally, its globular process about 1/6 length of signum.

#### Distribution.

China (Hainan).

#### Etymology.

The name is from the type locality.

## Supplementary Material

XML Treatment for Isodemis

XML Treatment for Isodemis illiberalis

XML Treatment for Isodemis stenotera

XML Treatment for Isodemis proxima

XML Treatment for Isodemis serpentinana

XML Treatment for Isodemis quadrata

XML Treatment for Isodemis guangxiensis

XML Treatment for Isodemis hainanensis

## References

[B1] Diakonoff A (1941) New Asiatic and Papuan Tortricidae with records of other species (3rd communication on Indo-Malayan and Papuan Microlepidoptera). Treubia 18:29-44

[B2] Diakonoff A (1948) Records and Descriptions of Microlepidoptera (2). Treubia 19 (3):483-524

[B3] Diakonoff A (1952) Wissenschaftliche Ergebnisse der Sumba-Expedition des Museums für Völkerkunde und des Naturhistorischen Museums in Basel, 1949. Microlepidoptera. Part 1. Verhandlungen der Naturforschenden Gesellschaft in Basel 63: 137–152

[B4] Diakonoff A (1976) Tortricidae from Nepal 2. Zoologische Verhandelingen, Leiden 144:1-145

[B5] Diakonoff A (1983) Tortricidea from Atjeh, Northern Sumatra (Lepidoptera). Zoologische Verhandelingen, Leiden 204: 1–132, pls 1–22.

[B6] Li HH (2002) The Gelechiidae of China (I) (Lepidoptera: Gelechioidea). Nankai University Press, Tianjin, 504 pp.

[B7] Meyrick E (1912) Tortricidae. In: Wagner (Ed) Lepidopterorum Catalogus. Pars 10. 8 6 pp.

[B8] Meyrick E (1918) Exotic Microlepidoptera 2(6): 161–192

[B9] Meyrick E (1921) New Micro-Lepidoptera. Zoologische Mededelingen, Leiden 6:145-202

[B10] Meyrick E (1922) Exotic Microlepidoptera 2(16): 481–512

[B11] Razowski J (2000) Tortricidae (Lepidoptera) collected in Taiwan with description of one new genus and eight new species, and a comparison with some regional faunas. Zoological Studies, 39 (4):319-327

[B12] Razowski J (2009a) Tortricidae from Vietnam in the Collection of the Berlin Museum. 5. Archipini and Sparganothini. SHILAP Revista de Lepidopterologia 37 (145):41-60

[B13] Razowski J (2009b) Tortricidae from Vietnam in the collection of the Berlin Museum. 7. Some additional data (Lepidoptera: Tortricidae). Polish Journal of Entomology, Institute Environmental Biology, Kazimierz Wielki University 78 (1):15-32

[B14] Walker F (1863) List of the specimens of Lepidopterous Insects in the collection of the British Museum. Order of the Trustees press, London 28:287-561

[B15] Walker F (1866) List of the specimens of Lepidopterous Insects in the collection of the British Museum. Order of the Trustees press, London 35:1535-2040

